# Energetic Al/Ni Superlattice as a Micro-Plasma Generator with Superb Performances

**DOI:** 10.1186/s11671-018-2795-8

**Published:** 2018-11-22

**Authors:** Yao Wang, Yichao Yan, Hongchuan Jiang, Zongren Xing, Yong Li, Wenzhi Qin, Liang Wang, Fei Guo

**Affiliations:** 1 State Key Laboratory of Electronic Thin Films and Integrated Devices, University of Electronic Science and Technology of China, Chengdu, 610054 China; 2 Institute of Chemical Materials, China Academy of Engineering Physics, Mianyang, 621900 China

**Keywords:** Al/Ni Superlattice, RMFs, Micro-plasma generator, Flyer velocity

## Abstract

In this study, energetic Al/Ni superlattice was deposited by magnetron sputtering. A micro-plasma generator was fabricated using the energetic Al/Ni superlattice. The cross-sectional micro-structure of the energetic Al/Ni superlattice was scanned by transmission electron microscopy. Results show that the superlattice is composed of Al layer and Ni layers, and its periodic structure is clearly visible. Moreover, the bilayer thickness is about 25 nm, which consists of about 15 nm Al layer and 10 nm Ni layer. The micro initiator was stimulated using a 0.22 μF capacitor charged at 2900–4100 V. The electrical behaviors were investigated by testing the current-voltage waveform, and the plasma generation was explored by ultra-high-speed camera and photodiode. The integrated micro generator exhibited remarkable electrical exploding phenomenon, leading to plasma generations at a small timescale. The plasma outputs reflected by flyer velocities were superior to that with a much thicker bilayer of 500 nm Al/Ni multilayer. The higher flyer velocity combined with Gurney energy model confirmed the chemical reaction of the Al/Ni superlattice structure contributed to plasma production in comparison with the Al/Ni multilayers. Overall, the energetic Al/Ni superlattice was expected to pave a promising avenue to improve the initiator efficiency at a lower energy investment.

## Introduction

Reactive multilayer foils (RMFs) contain stored chemical energy in the form of layer structures that undergo rapid energy release when stimulated by an external energy source [1–5]. The reaction velocity and temperature of these foils are closely related to the composition and geometry [6–9]. They are potential for materials welding [10–12], explosive initiation [13–15], and biological neutralization [16].

Among the numerous existing RMFs, Al/CuO [17], Al/MoO_3_ [18], Al/PTFE [19], B/Ti [20], and Al/Ni [21, 22] are most extensively studied. Al/Ni RMFs exhibits superiority due to their high reaction heat (330 cal/g), outstanding fabrication quality, and cost efficiency. Many works have been conducted to reveal the thermodynamics properties and the exothermic self-sustained reaction performances of Al/Ni RMFs [23–26]. Results implicates that the reaction performances (e.g., maximum combustion temperature, combustion delay time) of Al/Ni RMFs depend strongly on their bilayer thickness [27]. The RMFs with thinner bilayers have enhanced fuel/oxidizer interfacial contact areas and reduced average atomic diffusion distances so as to promote chemical reaction initiation [28]. Meanwhile, the reaction velocity and temperature increases as the bilayer thickness decreases. However, when the bilayer thickness of RMFs is below 20 nm, a contrary trend is found due to a large degree of intermixed region [29].

When the bilayer thickness of the Al/Ni RMFs is decreased to molecular or sub-nanometer scale, an energetic Al/Ni superlattice is formed. Energetic Al/Ni superlattice presents unique chemical reaction properties due to extremely short distances among reactants, and relatively large intermixed region. The chemical reaction of energetic Al/Ni superlattice was characterized by various methods (differential scanning calorimetry [29], transmission electron microscopy [30], and time-resolved X-ray microdiffraction [31]) to better understand the chemical reaction mechanism. Results indicated that metastable phase was not formed for superlattice structure due to its extremely low diffusion distance [32].

Extensive works have been carried out referring to the combustion characteristics and chemical mechanism of energetic Al/Ni superlattice. However, there are lack of reports on the electrical behaviors and plasma performances based on the energetic Al/Ni superlattice under extra electrical stimulation. In the present study, energetic Al/Ni superlattice was deposited on Al_2_O_3_ substrates by magnetron sputtering and patterned by wet etching to form a plasma generator. The electrical behaviors and plasma performance of the generator under electrical stimulation were investigated in detail.

## Experimental Methods

Energetic Al/Ni superlattice samples were fabricated by alternately depositing a layer composed of Al and Ni on Al_2_O_3_ substrates from Ni (99.99 wt%) and Al (99.99 wt%) targets. The base pressure of the deposition chamber was 5 × 10^−5^ Pa, and sputtering was performed with a process gas of Ar at pressures of 0.8 Pa. Both Al and Ni layer were deposited at 90 W. On above depositing conditions, the deposition rate for Al and Ni was about 15 nm/min and 10 nm/min, respectively. The bilayer thickness of as-deposited energetic Al/Ni superlattice was about 25 nm, and the overall thickness was about 4 μm. Each bilayer consisted of an Al layer and a Ni layer with a thickness ratio of 3:2 to maintain an overall 1:1 atomic ratio. As the comparison samples, Al/Ni RMFs with bilayer thickness of 500 nm were also deposited. The copper layer with thickness of 20 nm was deposited onto samples in order to keep good attachment to ceramic plug.

**Fig. 1:**
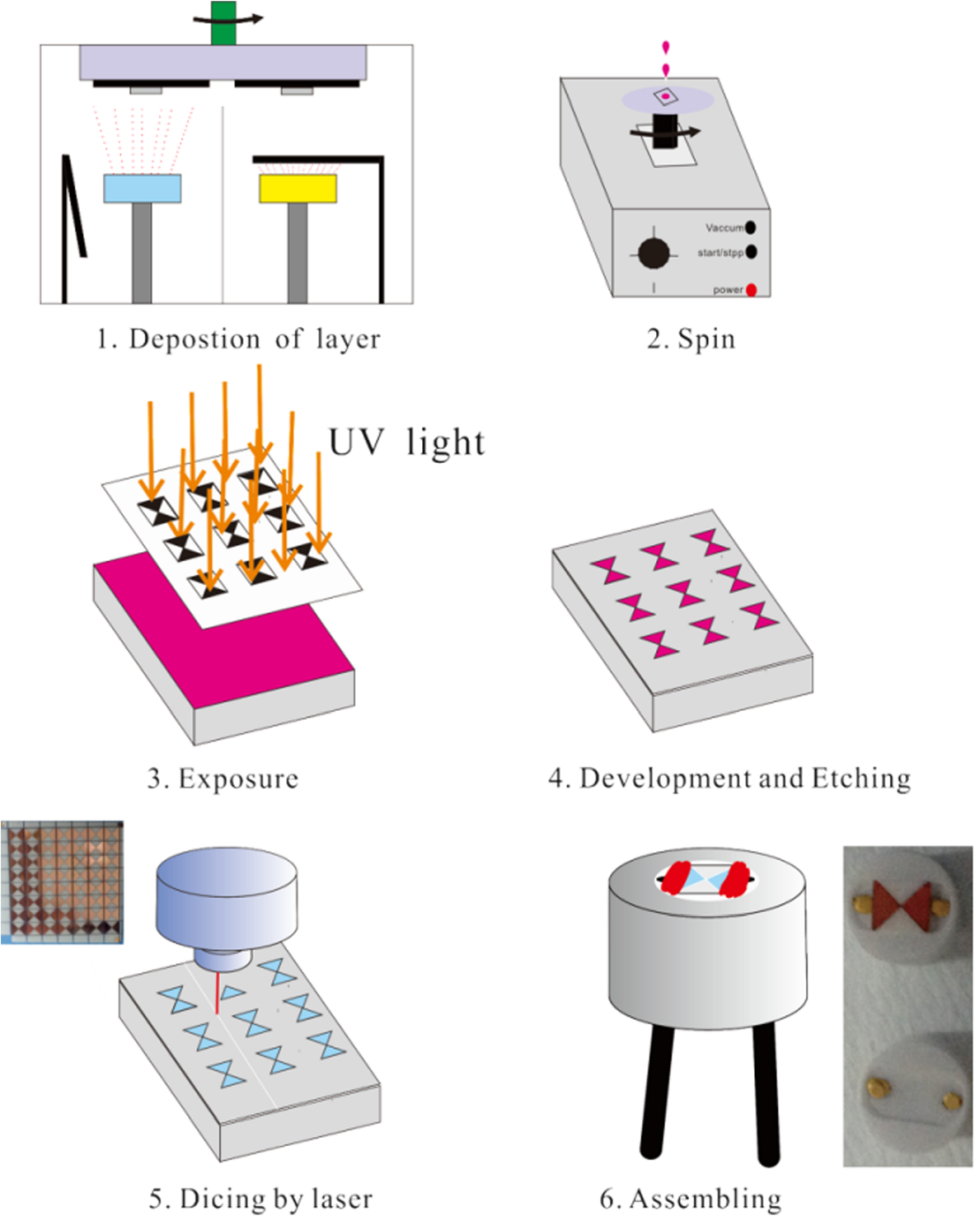
Fabrication process of micro plasma generator

The fabrication process of the micro-plasma generator was based on the MEMS technique, as shown in Fig. 1. Firstly, 0.5-mm-thick 4-in. Al_2_O_3_ substrate was cleaned with acetone, alcohol, and deionized water in an ultrasonic bath for 5 min, respectively. Secondly, the substrates were dried in an oven for 30 min at 100 °C. Thirdly, the substrates were fixed on the specimen holder and their surface contaminants were removed by oxygen plasma. Then, energetic Al/Ni superlattice was deposited on the surface of Al_2_O_3_ substrate. Subsequently, a positive photoresist (AZ5214E) was spin-coated on the surface of as-deposited samples at 5000 rpm for 60 s and pre-baked in an oven for 90 s at 100 °C. Afterwards, the samples were patterned and exposed to an ultraviolet radiation with an intensity of 16 mJ/cm^2^. Later, the samples were developed in NaOH solution. The samples were again baked at 120 °C to stabilize the photoresist pattern. Finally, the samples were etched to form a bowtie bridge in Al etchant solution (Aluminum Etchant Type A, Transene Company, Danvers, Massachusetts) at 30 °C. The patterned samples were diced into multiple individual chips, and the remaining photoresist was removed in acetone. Lastly, the chip was assembled into a ceramic plug to form the plasma generator.

**Fig. 2:**
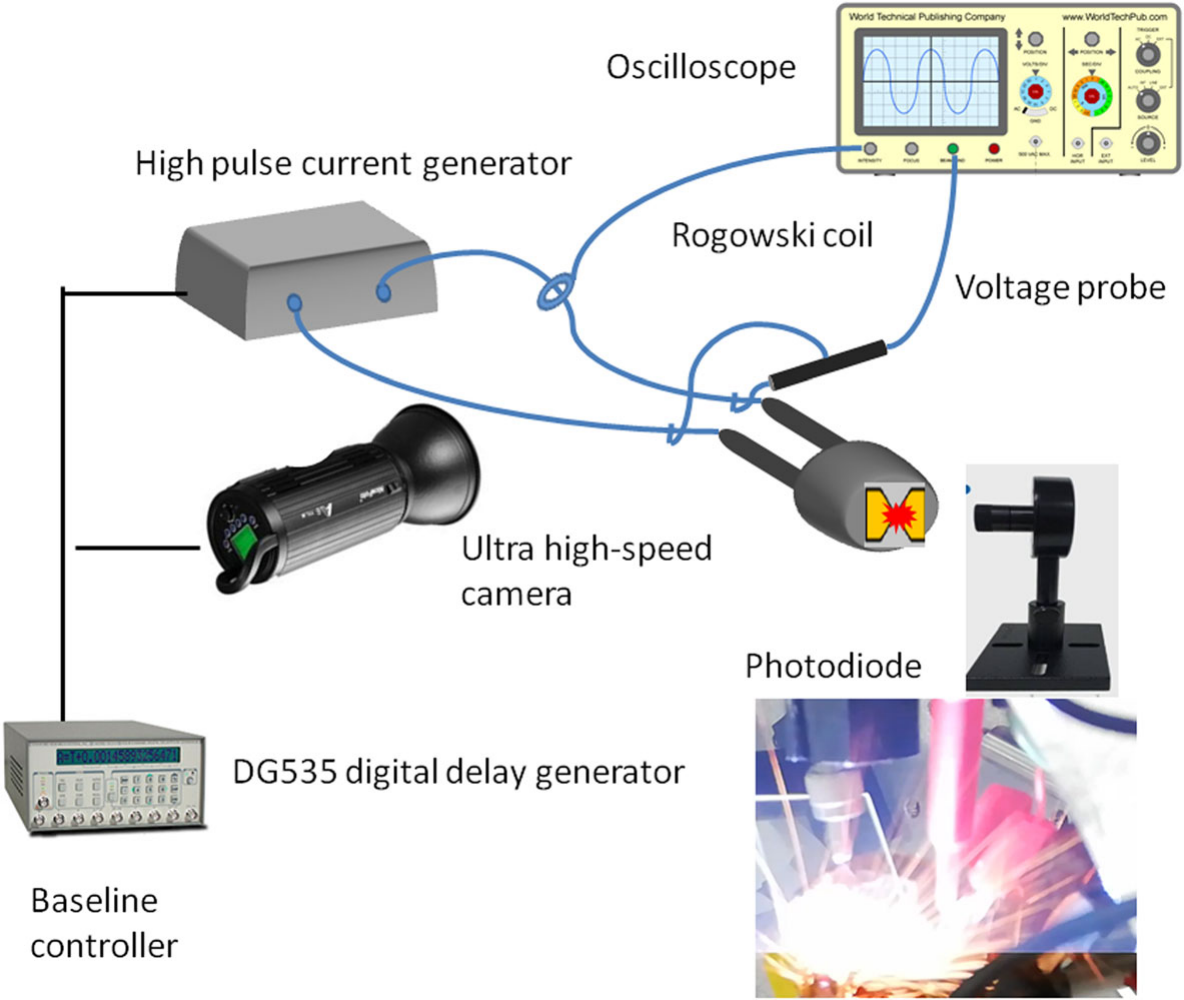
Testing schematic drawing of the micro-plasma generator

The cross-sectional structure of the plasma generator was characterized by the transmission electron microscopy (TEM). Subsequently, the micro-plasma generator was stimulated using a high-pulse current generator (0.22 μF, 2900–4100 V), and the current-voltage waveforms were measured using a Rogowski coil and a high-voltage probe, which recording by an oscilloscope. In the meantime, the plasma generation was recorded by a high-speed camera (SIM, SIL3001-00-H06). The exposure time of ultra-high-speed camera was 10 ns, and the interval time of each frame was about 20 –50 ns. In addition, the generating light intensity was measured by a photodiode. The testing baseline delay between the high-pulse current generator, ultra-high speed camera, and oscilloscope was controlled by a digital delay generator (DG535), which is shown in Fig. 2.

Furthermore, the performances of the micro-plasma generator were characterized by testing its ability to drive the Kapton flyer with the thickness of 30 μm. A short current pulse was applied on the plasma generator, causing a fast explosion of the bowtie bridge (0.4 × 0.4 mm), which in turn compelled the flyer to accelerate to a velocity up to several kilometer per second [33–35]. And the velocity of the flyer was recorded by a photonic Doppler velocimetry (PDV).

## Results and Discussion

**Fig. 3:**
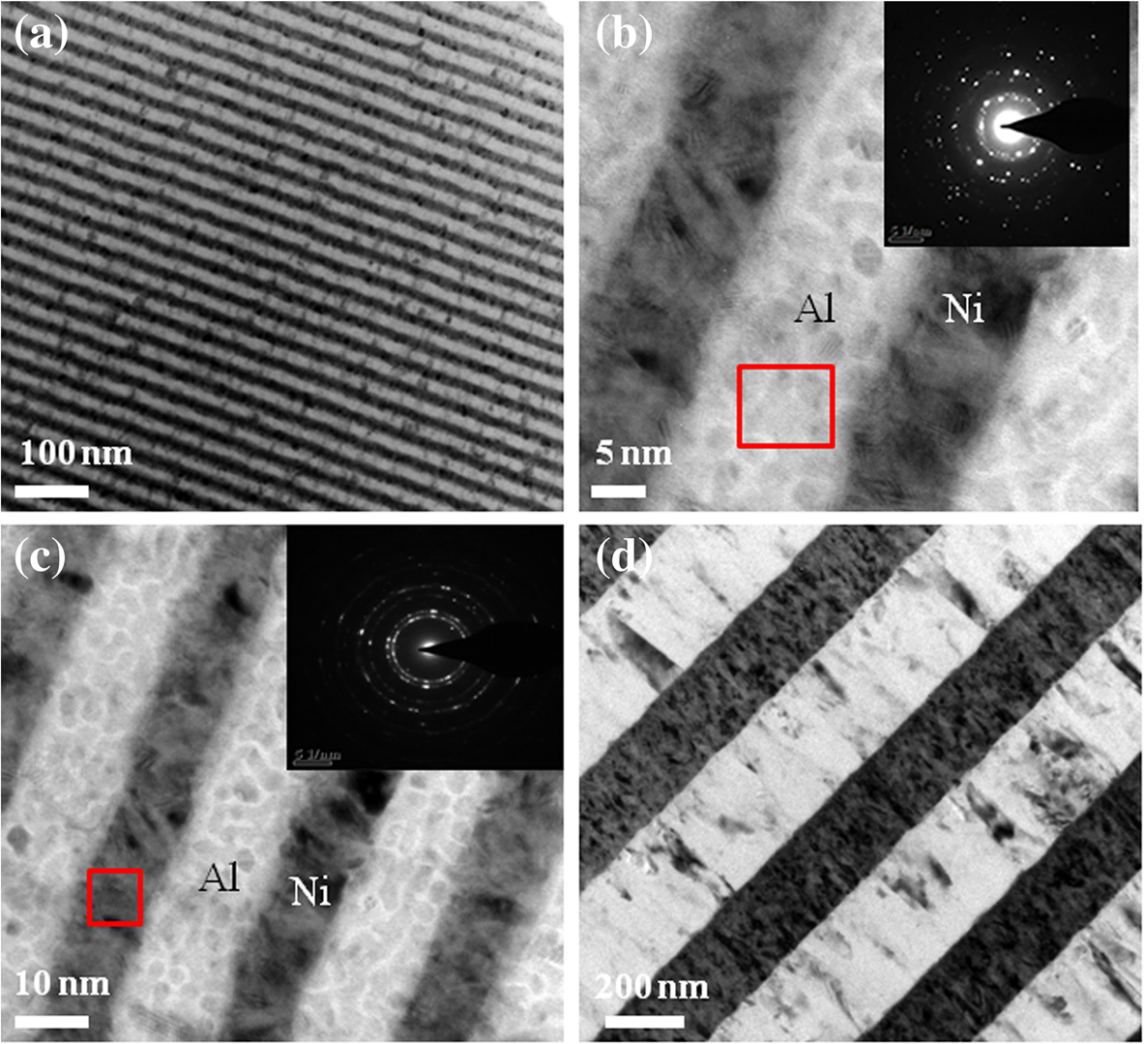
**a** Cross-sectional bright-field TEM image of the energetic Al/Ni superlattice. **b** Electron diffraction pattern of the Ni layer. **c** Electron diffraction pattern of the Al layer. **d** Cross-sectional bright-field TEM image of the Al/Ni RMFs

Figure 3a shows the cross-sectional bright-field TEM image of the energetic Al/Ni superlattice, which indicates a periodic structure consisting of Al and Ni bilayers with a controlled thickness, and different layers can be easily distinguished. The selected area electron diffraction (SAED) is further performed, as shown in Fig. 3b, c. The bright image corresponds to the Al layer, whereas the dark image denotes the Ni layer. The bilayer thickness is about 25 nm, which consist of about 15 nm Al layer and 10 nm Ni layer. The rings of diffraction indicate a well-defined polycrystalline structure of the Ni and Al layer. Figure 3d shows the cross-sectional bright-field TEM image of Al/Ni multilayers with a 500 nm bilayer thickness.

**Fig. 4:**
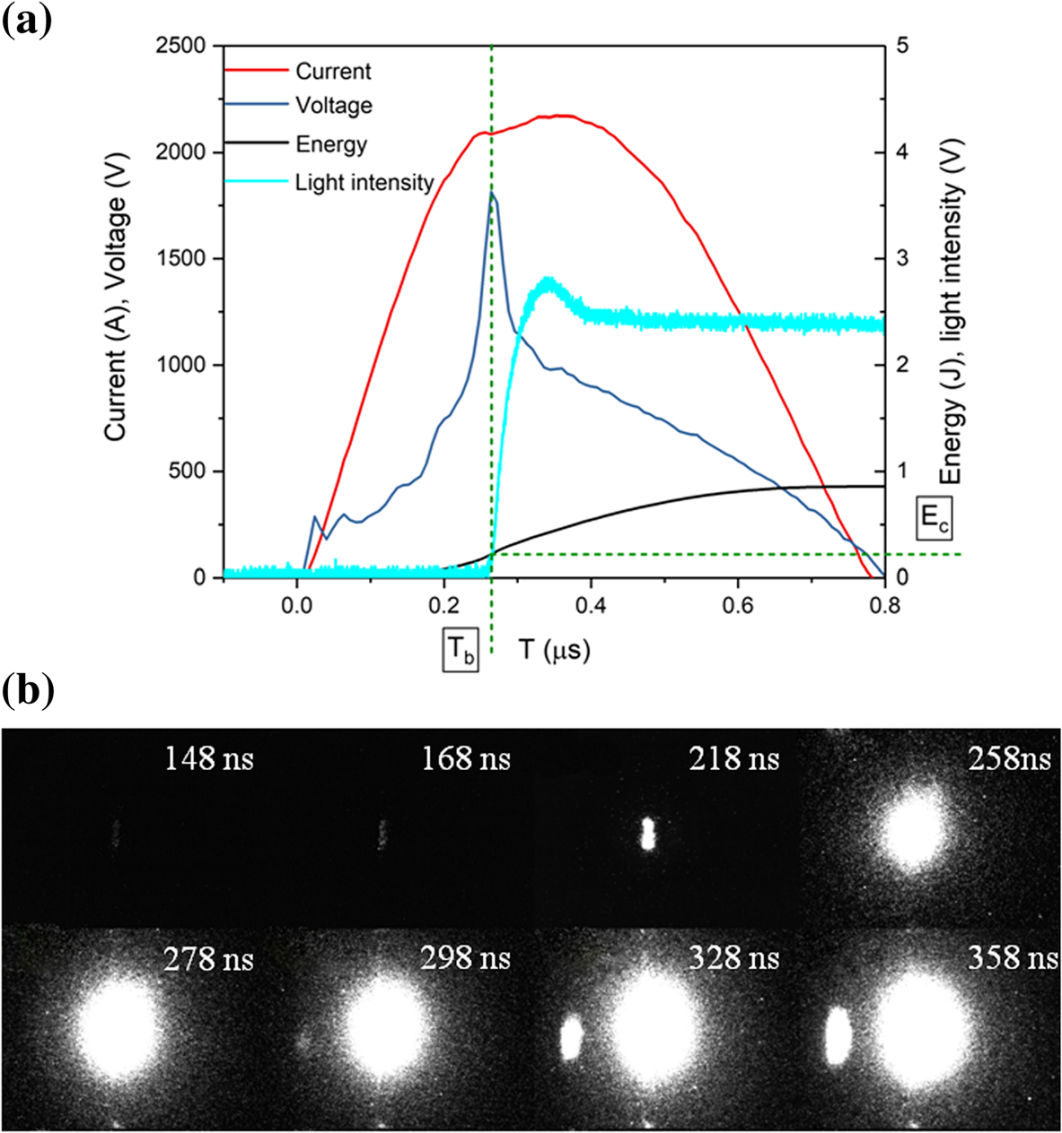
**a** Evolution of the current-voltage and light emission intensity for energetic Al/Ni superlattice with the storage capacitor initially charged 3.5 kV. **b** Cross-sectional images of the dynamic processes by ultra-high-speed camera

Figure 4a illustrates the voltage, current, light intensity, and energy histories of energetic Al/Ni superlattice charged at 3.5 kV. The evolution of voltage-current exhibits peaks of current and voltage. When a current pulse is supplied to the superlattice Al/Ni materials, the film is locally heated up due to the Joule effect, resulting in a rapid rise in the temperature corresponding to a rise in voltage across the bridge [36, 37]. Eventually, the voltage is enhanced highly enough to induce a drop in current, where the resistance reaches a maximum. The vaporized and ionized material constructs a new path of low resistance to make the voltage to drop towards zero accompanying with the discharging current to a maximum value.

Figure 4b shows the plasma evolution of energetic Al/Ni superlattice captured by ultra-high-speed camera. The process of Joule heating, evaporation, and plasma generating and expanding is evident. According to Fig. 4, a blurring light is observed, and the voltage and current rises slowly, indicating a Joule heating process (≤ 168 ns). At 218 ns, the voltage is suddenly increased while the emitting light is distinct, and the area of the light is nearly the area of the bowtie bridge. This corresponds to the evaporation process of the energetic Al/Ni superlattice. When the voltage reaches its maximum at 258 ns, the explosion associated with plasma generation takes place following with intense light. After exploding, the plasma expansion towards ambient is apt to cause shock wave. Product particles which existed in the combustion of Al/Ni RMFs are not observed in this study, implicating that the explosion of energetic Al/Ni superlattice is uniform under high-pulse current [38]. Therefore, the time of the voltage peak can be regarded as the delay time (*T*_b_) (between the onset of the current pulse and the voltage peak signal). The energy absorbed of the sample during this delay time is deemed as the critical explosion energy (*E*_c_). We should note that the point of onset light emission intensity corresponds to the voltage peak (258 ns). The signal of light emission intensity is hardly to be detected because of the weak light prior to explosion.

**Fig. 5:**
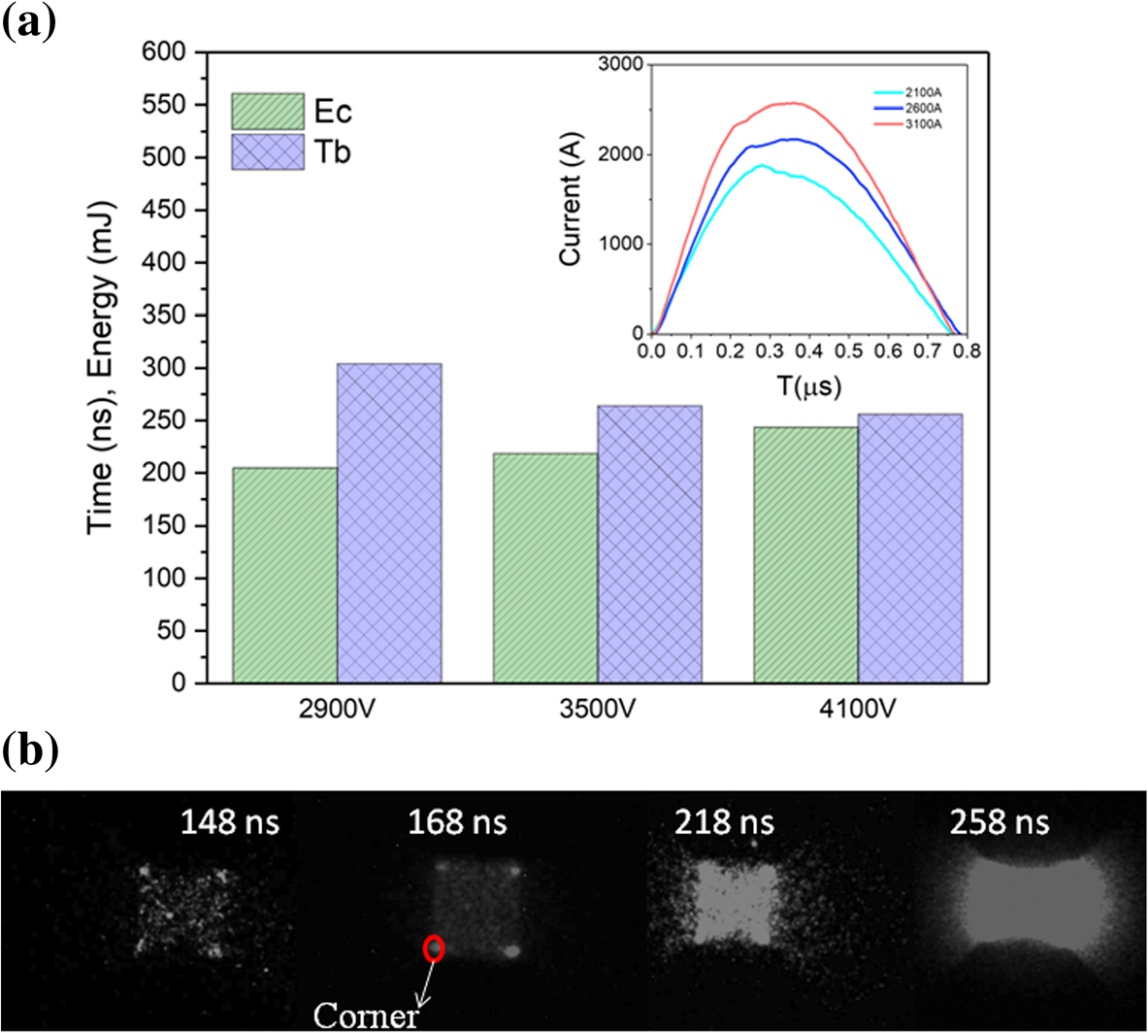
**a** Experimental results of the exploding time and critical explosion energy with charging voltages ranging from 2900 to 4100 V for energetic Al/Ni superlattice. **b** Images of the dynamic processes of energetic Al/Ni superlattice with the direction of towards the ultra-high-speed camera

The results of *T*_b_ and *E*_c_ are obtained from integrating electrical voltage-current curves under different charging voltage ranging from 2900 to 4100 V as presented in Fig. 5a. As shown in Fig. 5a, *T*_b_ decreases with the increase of charging voltage. According to the inset image in Fig. 5a, the maximum current reaches about 2572 A at 4100 V, whereas the current peak achieves 1870 A at 2900 V. It is indicated that the electrical energy input per unit time of energetic Al/Ni superlattice is increased with the increase of charging voltage. So, the delay time at lower charging voltage is much longer in comparison with high charging voltages. However, for *E*_c_ values, it exhibits an enhanced trend with the increase of charge voltage, implying that more electrical energy is absorbed to the point of exploding at 4100 V for energetic Al/Ni superlattice in comparison to that at 3500 and 2900 V, which can be ascribed to the exploding heterogeneity under an electrical pulse. When a high-current pulse is applied to energetic Al/Ni superlattice, the temperature of the four corners is much higher than that of other sections resulting in an explosion at a shorter time as shown on Fig. 5b. The difference between the exploding sections is diminished with increasing the charging voltage. Thus, the electrical exploding of energetic Al/Ni superlattice seems more homogeneous at 4100 V than those at lower charge voltages, accounting for the high exploding voltage and electrical energy.

**Fig. 6:**
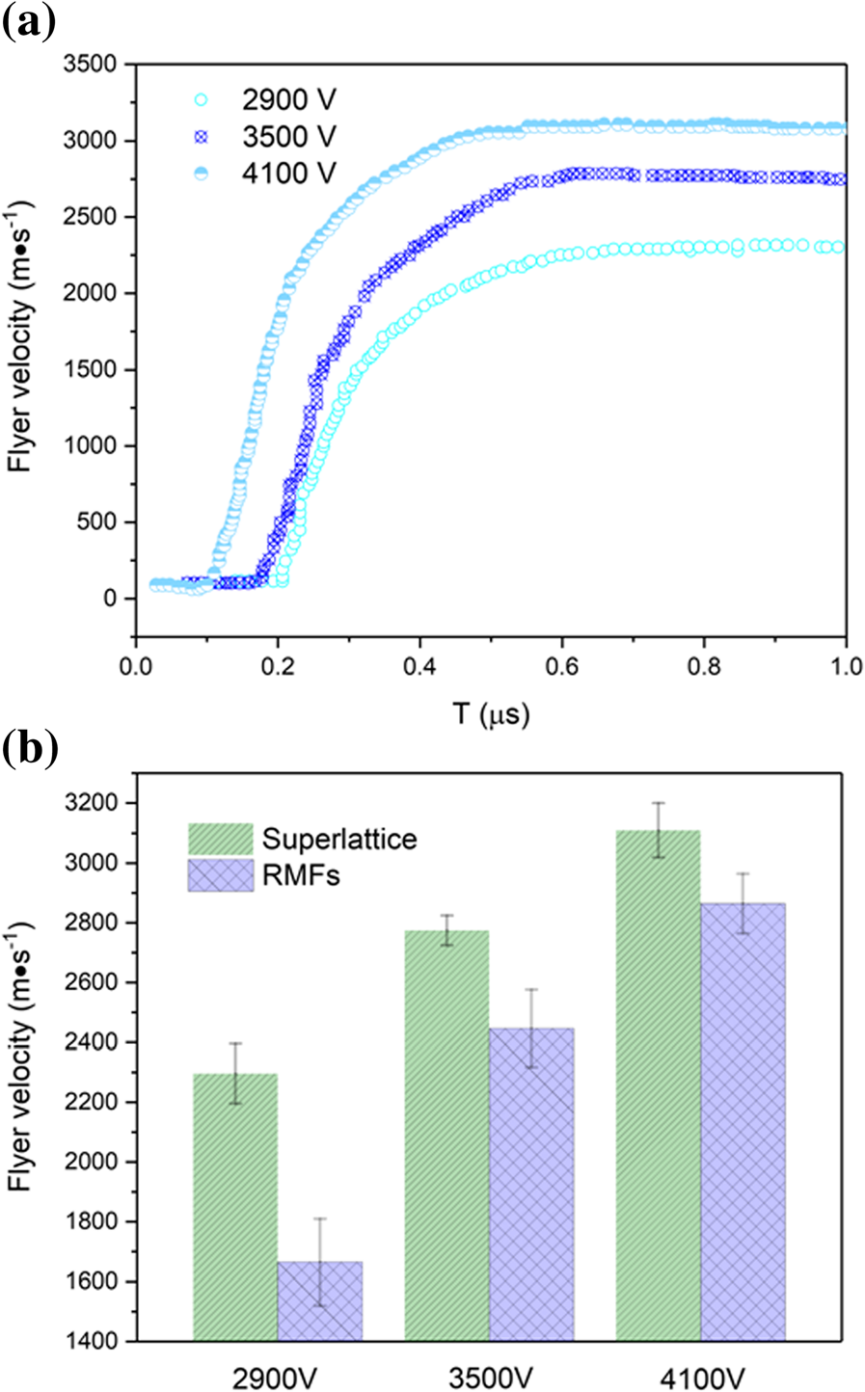
**a** Flyer velocity curves for different capacitor charging voltage levels applied to energetic Al/Ni superlattice. **b** Flyer velocity for the energetic Al/Ni superlattice and Al/Ni RMFs with charging voltages ranging from 2900 to 4100 V

Figure 6a shows the flyer velocities by plasma expansion at charging voltages ranging from 2900 to 4100 V for energetic Al/Ni superlattice. After the electrical pulse is loaded on the superlattice, the expanding plasma pressure accelerates the flyer away from the sample surface, causing a portion of the flyer to tear away and continue acceleration. As expected, the flyer velocity increases as the charging voltage is increased. For the charging voltage of 4100 V, the maximum flyer velocity achieves over 3 km/s, which is significantly higher than the peak value obtained at 3500 V charging voltage. When the charging voltage decreases to 2900 V, the flyer velocity is about 2.3 km/s.

The flyer velocity was measured three times at each charging voltage, and the maximum flyer velocity is averaged, as shown in Fig. 6b. Results show that the maximum flyer velocity of RMFs sample is much lower than that of energetic superlattice structures. Gurney energy model is introduced to reconcile different electrical energy and flyer-to-layers mass ratios between samples [39, 40]. The final flyer velocity is predicted according to:

1 $$ {v}_{\mathrm{f}}=\sqrt{2{E}_{\mathrm{g}}}{\left(\frac{M}{B}+\frac{1}{3}\right)}^{-\frac{1}{2}} $$

2 $$ {E}_{\mathrm{g}}=K{J_{\mathrm{b}}}^n $$

where *M* is the flyer mass, *B* is the mass from which the plasma energy for acceleration comes, and *E*_g_ is the energy per unit mass provided to the system. *K*, *n* is the Gurney factor which is decided by composition and geometry of foil. *J*_b_ is the electrical exploding current density. In the current case, the samples have the same flyer-to-layer mass ratios and Gurney factor due to the identical bilayer thickness, total thickness, and geometry. The flyer velocity is related to electrical energy provided to the system (*E*_g_), which is calculated by the exploding current density.

In our experiments results, the exploding current density of Al/Ni RMFs is higher than the superlattice. According to the Gurney energy model, the final flyer velocity of Al/Ni RMFs should exhibit a higher value in comparison to those samples integrated with energetic Al/Ni superlattice. But the predicted results are not consistent with experimental output (Fig. 6b). On the contrary, the experimental results with superlattice exhibit higher flyer velocity by contrasting RMFs. The increments of flyer kinetic energy confirm that the chemical energy generated from the reaction between Al and Ni is affected by the process of plasma for energetic Al/Ni superlattice. The heat release is attributed to high ionization of the superlattice during plasma formation process, resulting in a rapid plasma expansion velocity.

## Conclusions

In this work, energetic Al/Ni superlattice was fabricated by alternatively deposited Al and Ni layer on the surface of Al_2_O_3_ ceramic substrates by magnetron sputtering, characterized by TEM. The electrical behaviors and plasma performances based on the energetic Al/Ni superlattice under extra electrical stimulation were investigated, which was integrated as a micro-plasma generator. The integrated micro generator exhibited remarkable electrical exploding phenomenon, leading to plasma generations at a small timescale. The plasma outputs reflected by flyer velocities were superior to that with a much thicker bilayer of 500 nm Al/Ni multilayer. The Gurney energy model confirmed that the chemical reaction of the Al/Ni superlattice structure was involved in the plasma generation in comparison with Al/Ni RMFs. Overall, the micro plasma generator based on energetic Al/Ni superlattice exhibits superior performance with high-plasma outputs, thus improving electrical energy transduction and system reliability. Hence, there is much prospect of the application of energetic Al/Ni superlattice on micro- or nano-plasma initiators to implement special function.

## Abbreviations

RMFs: Reactive multilayer foils
TEM: Transmission electron microscopy
